# Validation of the Lithuanian multimorbidity treatment burden questionnaire (MTBQ) and its association with primary care patient characteristics

**DOI:** 10.1080/13814788.2023.2284257

**Published:** 2023-11-27

**Authors:** Olga Vasiliauskienė, Dovydas Vasiliauskas, Polly Duncan, Ausrine Kontrimiene, Lina Jaruseviciene, Aiste Cesnuleviciene, Gediminas Urbonas, Ida Liseckiene

**Affiliations:** a Department of Family Medicine, Lithuanian University of Health Sciences (LUHS), Kaunas, Lithuania; b Trinity University, San Antonio, TX, USA; c Centre for Academic Primary Care, NIHR School for Primary Care Research, University of Bristol, Bristol, UK

**Keywords:** Multimorbidity, treatment burden, mTBQ, validation, primary health care, lithuania

## Abstract

**Background:**

The increasing prevalence of multimorbidity among older people in Lithuania and other Central-Eastern European countries leads to a greater patient treatment burden and puts additional pressure on healthcare services.

**Objectives:**

This study aimed to validate the Lithuanian version of the Multimorbidity Treatment Burden Questionnaire (MTBQ).

**Methods:**

The Lithuanian version of the MTBQ was tested (2021-2022) with 789 patients from seven Lithuanian primary care centres who had two or more long-term conditions. The questionnaire translation’s reliability, validity and dimensionality of the were assessed with Spearman’s rank correlation, Cronbach’s alpha, and factor reduction analysis. Treatment burden and its associations with sociodemographic and other indicators were analysed.

**Results:**

Lithuanian version of MTBQ had good internal reliability (Cronbach’s alpha 0.711), validity, factor reduction applicability, and interpretability. The MTBQ scores of the questionnaire had a negative association with the quality-of-life scale (r=-0.327, 95% CI [-0.389, −0.264]) and positive associations with the self-rated health scores (*r* = 0.230, 95% CI [0.163, 0.297]) and with the number of comorbidities (*r* = 0.164, 95% CI [0.097, 0.233]). Distribution of treatment burden was identified (none (19,4%), low (46,6%), medium (25%), high (9%)). High treatment burden was found to be associated with having five or more long-term diseases, taking five or more medications, having anxiety or depression and living in a rural area.

**Conclusion:**

The study’s findings show that the MTBQ is applicable in assessing the treatment burden of multimorbid patients in Lithuania. Furthermore, the study demonstrates that Lithuanian patients with multimorbidity have average treatment burden scores similar to or higher than participants in previous MTBQ validation studies.

## KEY MESSAGES

• The Lithuanian version of MTBQ features good content validity, internal reliability, and construct validity, and is suitable for assessing treatment burden of patients with multimorbidity in Lithuania.

• The study demonstrated that Lithuanian patients with multimorbidity had average treatment burden scores similar to or higher than participants in previous MTBQ validation studies.

## Introduction

Multimorbidity (having two or more chronic health conditions) has become prevalent in ageing populations worldwide [1], including Lithuania, where more than 50% of 65–75-year-old patients have more than two long-term conditions and the average number of long-term conditions increases every year [2, 3]. Healthcare recommendations and support are usually focused on patients with a single chronic disease [4], therefore, patients with multimorbidity typically have to independently handle their healthcare responsibilities, such as frequent appointments with primary care professionals and other specialists, regular intake of multiple medications, self-monitoring of medical conditions and other self-care tasks [2, 3]. Altogether, these patient responsibilities can become overwhelming, hinder treatment success, harm the quality of patient’s life, and increase the risk of hospitalisation and death [5, 6].

The difficulties related to treatment and self-management of chronic health conditions and their effects on patient functioning and well-being are regarded as a treatment burden [7]. Treatment burden evaluation tools can help determine the effectiveness of the healthcare system in responding to the needs of patients with multiple long-term conditions [8]. Recently, several such tools have been developed [9–11]; however, none have yet been validated and adapted for the Lithuanian population. Our study proposes the Lithuanian version of the Multimorbidity Treatment Burden Questionnaire (MTBQ) as the first treatment burden evaluation tool adapted for the Lithuanian population.

The original MTBQ [12] and its population-adapted translations have demonstrated reliability and construct validity [13–16]. The questionnaire covers a broad range of treatment burden factors (medications, lifestyle changes, appointments with multiple physicians, and independence of disease monitoring) that are all relevant in Lithuanian healthcare [2]. Multimorbidity affects one-sixth of the Lithuanian adult population and corresponds to a multifold increase in home visits, 30-day hospital readmission rates and higher healthcare costs [3]. The MTBQ is expected to contribute to a better understanding and evaluation of treatment burden which might be instrumental in further studies and actions aiming to improve the healthcare quality of patients with multimorbidity in Lithuania [17, 23].

In this study, we aim to validate the Lithuanian version of Multimorbidity Treatment Burden Questionnaire (MTBQ) for patients with multimorbidity and identify factors associated with high treatment burden in the Lithuanian population.

## Methods

### Study setting

The study was conducted at seven Lithuanian primary healthcare centres (five urban, two rural) in Kaunas, Šiauliai and Tauragė municipalities between the years 2021–2022 as a part of the TELELISPA Project ‘Improved Healthcare Quality for Patients with Multimorbidity in Lithuania’ (Project number 08.4.2-ESFA-K-616-01-0003). The project aims to improve the healthcare management of people with multimorbidity by implementing a novel healthcare model in Lithuanian primary healthcare centres that include case managers, a multidisciplinary team approach and patient-centred personalised healthcare.

### Translation

The MTBQ was translated into Lithuanian following the 4-step process from previous MTBQ validation studies [13–16]. The original English MTBQ version was first translated into Lithuanian by a professional native Lithuanian translator, bilingual in English and Lithuanian. The initial translation was then reviewed by three Lithuanian healthcare experts with English proficiency and back-translated to English by an English translator. The back-translated version was reviewed again and compared with the original English version by a group of English-proficient healthcare experts.

### Patient and public involvement

A pilot group of 15 patients was randomly selected for a pre-test of the Lithuanian MTBQ translation to evaluate the comprehensibility of the questions. The patients were selected through the primary sample collection at the beginning of the project. The patients completed the questionnaire and discussed the wording clarity and layout in face-to-face interviews with the healthcare experts.

### Data collection

This study involved all participants of the TELELISPA project (*n* = 796). The participants were 40 to 85 years of age and had two or more long-term health conditions. They completed the Lithuanian versions of the MTBQ and quality-of-life EQ-5D-5L questionnaires, answered questions about their sociodemographic status, and self-rated their health. The MTBQ score was calculated for participants (*n* = 789, 99.1%) who answered at least 75% of the MTBQ questions.

### Scoring of the questionnaires

Each of the question items in MTBQ translation questions was scored on a 5-point scale as follows: 0 (not difficult or does not apply), 1 (a little difficult), 2 (quite difficult), 3 (very difficult), and 4 (extremely difficult). All question items with a high proportion of ‘does not apply’ scores (> 5%) were omitted from the final version of the questionnaire following the previous MTBQ validation studies [12, 13], and the overall MTBQ score of the shortened MTBQ questionnaire was calculated following the original MTBQ scoring instructions [12]. The partial proportional odds analysis was conducted across four treatment burden groups – no burden, low burden, medium burden, and high burden – following the previous MTBQ validation studies [12–13].

The MTBQ scores were compared with the scores of the quality-of-life EQ-5D-5L questionnaire and self-rated health scores [18]. The participants’ answers for each of the five EQ-5D-5L questionnaire categories were weighted on a 5-point scale and re-expressed into EQ-5D-5L index value using the European EQ-5D-5L value sets. The self-rated patient health scores were obtained from patient responses to a single-item question (‘How would you rate your health?’) on a 5-point scale (1-excellent, 5-poor).

### Sociodemographic data

The sociodemographic survey included the following factors: employment status, education level and residential area (urban or rural). Education levels were grouped in terms of the Lithuanian education structure, and employment status was grouped into three categories (employed/unemployed/permanently out of work).

### Long-term conditions and medications

All study participants had two or more long-term health conditions, one of which had to be arterial hypertension. The participants were asked to self-report having any long-term conditions listed among the 12 most prevalent long-term disorders in Lithuania and any other long-term conditions [2]. The number of regularly taken medications was then recorded from self-reported data and medical records with the permission of the patient and patients’ family practice institutions.

### Statistical analysis

Descriptive statistics were generated for the study population. Data analysis of sociodemographic factors, long-term conditions, and treatment burden questionnaires was conducted using IBM SPSS V29.0. The data were analysed regarding reliability, validity, translation quality, and score interpretability proposed by the International Society for Quality-of-Life Research (ISOQOL) [19].

### Reliability

The internal consistency reliability of the Lithuanian version of MTBQ was examined by calculating Cronbach’s alpha and inter-item correlation matrix per previous MTBQ validation studies [12–16]. Cronbach’s alpha values of 0.7-0.9 and interitem correlation values of 0.15-0.50 were deemed ideal for the data consistency and reliability analysis [22, 23].

### Construct validity

Construct validity of the Lithuanian MTBQ translation was tested by examining the relationship between the global score of MTBQ questionnaire values (0 – minimal burden, 100 – maximum burden), the EQ-5D-5L, the self-rated health score, and the self-reported number of comorbidities. A valid Lithuanian MTBQ translation was expected to have a negative association between the global MTBQ score and EQ-5D-5L score and positive associations between the global MTBQ score and self-rated health score as well as an increase in the number of long-term diseases (*p* < 0.05) [12].

### Dimensionality

The dimensionality of the items in the Lithuanian version of MTBQ was assessed through factor reduction analysis. The sampling adequacy and the appropriateness for factor reduction were evaluated by the Kaiser-Meyer-Olkin Test (values between 0.7 and 1 deemed acceptable) [20], Bartlett’s Test of Sphericity (acceptable *p* < 0.05) [21], and Scree plot [12]. The questionnaire items were grouped into factors if they had acceptable factor loading values of at least 0.40.

### Responsiveness

The responsiveness was not assessed as the study was based on cross-sectional data.

### Interpretability of scores

The global MTBQ treatment burden values were grouped into four categories: no burden (score of 0), low burden (score < 10), medium burden (score of 10–22), and high burden (score ≥ 22) used in the previous MTBQ validation studies [12, 13]. The associations between the treatment burden category levels and other data (sex, age, education, number of long-term conditions, number of medications, anxiety level, depression level, and long-term conditions) were analysed through partial proportional odds models at a 5% significance level.-

### Ethical approval and data sharing

The study was registered by Lithuanian Bioethics Committee (permission no. L-21-03/5) under the TELELISPA01 (V1.4) protocol as part of the TELELISPA project. Each participant received a written form about the purpose of the study and gave informed consent for their participation. All methods were carried out under relevant guidelines and regulations. The respondents‘data have been depersonalised.

## Results

### Participants

The sample of study participants (*n* = 789) had a mean age of 64.4 years, with a slight majority of women (61%). Almost half of the participants were permanently retired (47.9%), and approximately two-fifths were employed (38.1%). About two-thirds of the participants had tertiary education, with either a professional (34.9%) or a university (28.4%) degree. About one-third of the participants had a mild or higher level of anxiety (28.6%) and depression (33.8%). Slightly more than half (54.3%) of the participants had 2-4 long-term health conditions (Table 1).

**Table 1.** Participant characteristics (*n* = 789).

	*N*	%
Mean age (SD), years	64.38 (9.7)	
Age years (missing data: *n* = 38, 4.8%)		
< 60	223	28.3
60–64	174	22.1
65–70	120	15.2
70+	234	29.7
Gender (missing data: *n* = 0; 0%)		
Male	308	39.0
Female	481	61.0
Educational level (missing data: *n* = 6, 0.7%)		
Primary education (0–10 years)	185	23.4
High school (11–12 years)	99	12.5
Some tertiary education (13–14 years)	275	34.9
University (14+ years)	224	28.4
Employment status (missing data: *n* = 3, 0.3%)		
Employed	301	38.1
Unemployed	72	9.1
Employed in retirement	59	7.5
Retired	319	40.4
Other	35	4.4
Number of self-reported long-term conditions^a^ (missing data: *n* = 1, 0.1%)		
2	65	8.2
3	216	27.4
4	147	18.7
5	108	13.7
> 5	252	32.0
Number of medications (missing date: *n* = 0; 0%)		
0-4	245	31.1
> 5	544	68.9
Anxiety level (GAD-7 scale) (missing data: *n* = 2, 0.3%)		
Minimal (0–4)	561	71.1
Mild or higher (5–21)	226	28.6
Depression level (PHQ-9 scale)		
Minimal (0–4)	522	66.2
Mild or higher (5–27)	267	33.8
Area of residence		
Urban	671	85.0
Rural	118	15.0
Health measure scores		
Mean quality of life score (SD)^b^		0.74 (0.19)
Mean self-reported health score (SD)^c^		3.62 (0.69)
Mean number of self-reported comorbidities (SD)		2.68(1.20)

^a^ Number of self-reported conditions including any of the 12 most common long-term conditions in Lithuania that were listed in the survey [2], and any additional self-reported conditions. Conditions within a cluster of similar risk factors and treatment methods (for example, asthma and COPD) were only counted once.

^c^ Score based on EQ-5D-5L index score [17].

^c^ Score based on the answers to a single question ‘How would you rate your health?:’ Excellent (1), Very good (2), Good (3), Fair (4), Poor (5).

### Question properties

Each question item of the back-translated questionnaire was deemed to retain the essential information by the healthcare expert group. The 15 patients who participated in the original pilot testing of the questionnaire took approximately ten minutes to complete the Lithuanian version of MTBQ and found it easy to understand. Consequently, no further changes were made to the back-translated questionnaire.

Similarly to the previous MTBQ validation studies, the questionnaire responses were positively skewed and had high floor effects (added proportion of ‘does not apply’ and ‘no burden’ responses) ranging between 55% and 91% [12–16]. The global MTBQ scores were also positively skewed, with a floor effect (global score of zero) of 18.5%. None of the questions had a missing data proportion higher than 0.3% (Table 2).

**Table 2.** Responses to the Multimorbidity Treatment Burden Questionnaire (*n* = 789). question items with a large percentage of ‘does not apply’ responses (> 5%) are highlighted in bold.

Please tell us how much difficulty you have with the following:	Not difficult *n* (%)	A little difficult *n* (%)	Quite difficult *n* (%)	Very difficult *n* (%)	Extremely difficult *n* (%)	Does not apply *n* (%)	Missing data *n* (%)
1. Taking lots of medications	497 (63.0)	173 (21.9)	69 (8.7)	16 (2.0)	4 (0.5)	30 (3.8)	0 (0)
2. Remembering how and when to take medication	583 (73.9)	152 (19.3)	26 (3.3)	10 (1.3)	6 (0.8)	11 (1.4)	1 (0.1)
3. Paying for medications and treatment	640 (81.1)	96 (12.2)	31 (3.9)	10 (1.3)	6 (0.8)	6 (0.8)	0 (0)
4. Collecting prescription medication	718 (91.0)	49 (6.2)	9 (1.1)	1 (0.1)	0 (0)	12 (1.5)	0 (0)
5. Monitoring your medical conditions	662 (83.9)	87 (11.0)	21 (2.7)	5 (0.6)	4 (0.5)	10 (1.3)	0 (0)
6. Arranging appointments with health professionals	579 (73.4)	129 (16.3)	45 (5.7)	27 (3.4)	5 (0.6)	4 (0.5)	0 (0)
7. Seeing lots of different health professionals	422 (53.5)	190 (24.1)	106 (13.4)	47 (6.0)	10 (1.3)	14 (1.8)	0 (0)
8. Getting time off work, arranging transport, etc. to see doctors	516 (65.4)	143 (18.1)	62 (7.9)	35 (4.4)	9 (1.1)	24 (3.0)	0 (0)
9. Getting health care in the evenings and at weekends	321 (40.7)	98 (12.4)	33 (4.2)	8 (1.0)	1 (0.1)	**326 (41.3)**	2 (0.3)
10. Getting help from community services	323 (40.9)	86 (10.9)	31 (3.9)	3 (0.4)	5 (0.6)	**341 (43.2)**	0 (0)
11. Obtaining clear and up-to-date information about your condition	691 (87.6)	54 (6.8)	15 (1.9)	2 (0.3)	0 ()	27 (3.4)	0 (0)
12. Making recommended lifestyle changes	399 (50.6)	209 (26.5)	115 (14.6)	34 (4.3)	13 (1.6)	19 (2.4)	0 (0)
13. Having to rely on help from family and friends	693 (87.8)	49 (6.2)	19 (2.4)	1 (0.1)	2 (0.3)	25 (3.2)	0 (0)

Questions 9 and 10 were found to have remarkably high ‘does not apply’ response proportions (41% and 43%) and were consequently excluded from the final analysis (the ‘does not apply’ response proportion did not exceed 4% for any other question). The exclusion of the two questions did not significantly affect dimensionality, reliability, and validity analysis results.

### Reliability

The reduced 11-item questionnaire had Cronbach’s alpha value of 0.711, within the acceptable range of 0.7-0.9. Most items fell within the ideal inter-item correlation range (*r* = 0.15–0.50). A high inter-item correlation was found between questions 6, 7 and 8 (with respective correlations of 0.626, 0.627 and 0.621). The Cronbach alpha and inter-item correlation analysis indicated satisfactory reliability of the Lithuanian MTBQ translation.

### Construct validity

The global MTBQ score had a negative association with the index score of the EQ-5D-5L questionnaire (r=-0.327, *p* < 0.001), a positive association with the self-rated health score (*r* = 0.230, *p* < 0.001), and a positive association with the number of comorbidities (*r* = 0.164, *p* = < 0.001) (Table 3).

### Dimensionality

The reduced 11-item MTBQ version had Kaiser-Meyer-Olkin test value of 0.773 and Bartlett’s test of sphericity significance of *p* < 0.001, which indicated that sampling was adequate and that the MTBQ question translations were suitable for the reduced factor analysis. The Scree plot suggested a three-factor solution. According to the rotated component matrix analysis (Varimax with Kaiser normalisation), the questions were reducible to 3 component groups (Table 4). Parallel eigenvalue analysis showed that a factor with the highest factor-item correlation explained 27.3% of the total variance.

### Interpretability of scores

The results suggest that after adjusting for age, the patients had significantly higher odds of an increased treatment burden if they had more than five long-term conditions, regularly took more than five medications, lived in a rural area, had anxiety or depression, a history of heart failure or long-term kidney disease, a lower-than-maximum EQ-5D-5L index score, a lower-than maximum self-rated health score, and four or more long term diseases (Table 5). No significant differences in treatment burden odds were observed across age, gender, and education level groups.

**Table 3.** Association between global MTBQ score and other health and life quality measurement tools (EQ-5D-5L index score, self-rated health score, and the number of comorbidities).

Variable	Spearman’s rank correlations (r_s_)	*P-*values
EQ-5D-5L Index Score^a^	−0.327	**< 0.001**
Self-rated health^b^	0.230	**< 0.001**
Number of comorbidities^c^	0.164	**< 0.001**

^a^ Based on EQ-5D-5L index score [17].

^b^Score based on the answers to a single question ‘How would you rate your health?:’Excellent (1), Very good (2), Good (3), Fair (4), Poor (5).

^c^Number of self-reported conditions including hypertension, any of the 12 conditions that were listed in the survey, and any additional self-reported conditions. Conditions within a cluster of similar risk factors and treatment methods (for example, asthma and COPD) were only counted once.

**Table 4.** Factor reduction analysis results for the study population (*N* = 789) obtained from rotated component matrix with minimum factor loading values of 0.4.

	Factor 1	Factor 2	Factor 3
Q6	.854		
Q7	.839		
Q8	.850		
Q3		.454	
Q4		.449	
Q5		.526	
Q11		.483	
Q12		.598	
Q13		.649	
Q1			.783
Q2			.808

**Table 5.** Partial proportional odds analysis for treatment burden within different patient groups (*n* = 789).

	Treatment burden score						
	None (0)	Low (1–9)	Medium (10–21)	High (≥ 22)	Unadjusted OR^a^	Adjusted OR^b^	*P*-value
Participants (%)	153 (19.4)	368 (46.6)	197 (25.0)	65 (9.0)			
Mean age, years (SD)	65.3 (9.7)	64.5 (9.8)	63.8 (9.5)	63.19 (9.7)	**0.988 (0.974 to 1.002)**	0.988 (0.974 to 1.002)	0.082
Gender (*n*, (%))							
Female (ref.)	102 (21.2)	220 (45.7)	113 (23.5)	46 (9.6)			
Male	51 (16.6)	148 (48.1)	84 (27.3)	25 (8.1)	1.190 (0.910 to 1.555)	1.130 (0.856 to 1.491)	0.204
Number of long-term conditions (*n*, (%))^c^							
2–4 (ref.)	106 (24.7)	204 (47.6)	98 (22.8)	21 (4.9)			
5+	47 (13.1)	164 (45.6)	99 (27.5)	50 (13.9)	**1.949 (1.492 to 2.546)**	**2.037 (1.531 to 2.711)**	**< 0.001**
Number of medications (*n*, (%))^d^							
0–4 (ref.)	58 (23.7)	120 (49.0)	52 (21.2)	15 (6.1)			
5+	95 (17.5)	248 (45.6)	145 (26.7)	56 (10.3)	**1.518 (1.143 to 2.017)**	**1.532 (1.141 to 2.058)**	**0.004**
Long-term conditions (*n*, (%))^e^							
Cardiovascular Disease (I20)	58 (18.8)	143 (46.3)	80 (25.9)	28 (9.1)	1.072 (0.820 to 1.402)	1.073 (0.810 to 1.422)	0.611
Long-term Ischaemic Heart Disease (I25)	12 (15.4)	38 (48.7)	20 (25.6)	8 (10.3)	1.175 (0.760 to 1.816)	1.307 (0.836 to 2.045)	0.241
Heart Failure (I50)	30 (12.9)	114 (49.1)	58 (25.0)	30 (12.9)	**1.444 (1.084 to 1.924)**	**1.486 (1.105 to 1.999)**	**0.009**
Atrial Fibrillation (I48)	21 (18.8)	50 (44.6)	29 (25.9)	12 (10.7)	1.111 (0.762 to 1.620)	1.189 (0.801 to 1.763)	0.390
Diabetes (E11)	46 (15.9)	137 (47.2)	77 (26.6)	30 (10.3)	1.298 (0.989 to 1.703)	1.184 (0.896 to 1.565)	0.060
Thyroid Gland Diseases (E06.3)	16 (19.8)	42 (51.9)	16 (19.8)	7 (8.6)	0.835 (0.545 to 1.277)	0.793 (0.515 to 1.220)	0.291
Hypothyroidism (E89)	6 (23.1)	8 (30.8)	10 (38.5)	2 (7.7)	1.346 (0.622 to 2.914)	1.345 (0.595 to 3.042)	0.451
Asthma or COPD (J44)	6 (22.2)	8 (29.6)	9 (33.3)	4 (14.8)	1.472 (0.688 to 3.152)	1.430 (0.644 to 3.177)	0.751
Asthma (J45)	14 (18.4)	27 (35.5)	25 (32.9)	10 (13.2)	1.535 (0.970 to 2.428)	1.470 (0.912 to 2.367)	0.067
Joint Disease (M05M06)	5 (17.2)	13 (44.8)	9 (31.0)	2 (6.9)	1.185 (0.589 to 2.383)	1.108 (0.544 to 2.255)	0.734
Osteoporosis (M80M81)	4 (14.3)	10 (35.7)	9 (32.1)	5 (17.9)	1.868 (0.898 to 3.884)	1.912 (0.902 to 4.050)	0.091
Long-term kidney disease (N18)	8 (16.3)	15 (30.6)	18 (36.7)	8 (16.3)	**2.034 (1.148 to 3.604)**	**2.129 (1.188 to 3.815)**	**0.011**
GAD-7 anxiety score (*n*, (%))^f^							
Low (0–4)	128 (22.8)	276 (49.2)	127 (22.6)	30 (5.3)			
High (5–21)	25 (11.1)	92 (40.7)	69 (30.5)	40 (17.7)	2.392 (1.775 to 3.225)	2.395 (1.762 to 3.255)	< 0.001
PHQ-9 depression score (*n*, (%))^g^							
Low (0–4)	119 (22.8)	266 (51.0)	114 (21.8)	23 (4.4)			
High (5–27)	34 (12.7)	102 (38.2)	83 (31.1)	48 (18.0)	**2.502 (1.877 to 3.334)**	**2.520 (1.894 to 3.352)**	**< 0.001**
Education level (*n*, (%))							
0–13 years (ref.)	107 (19.1)	257 (45.9)	142 (25.4)	54 (9.6)			
14+ years	44 (19.6)	109 (48.7)	54 (24.1)	17 (7.6)	0.900 (0.673 to 1.202)	1.011 (0.750 to 1.363)	0.475
Residential area							
Urban (ref.)	139 (20.7)	318 (47.4)	168 (25.0)	46 (6.9)			
Rural	14 (11.9)	50 (42.4)	29 (24.6)	25 (21.2)	**1.833 (1.263 to 2.659)**	**1.869 (1.281 to 2.726)**	**0.001**
Scores of health measures^h^							
Mean self-rated health score (SD)^i^	3.35 (0.71)	3.64 (0.62)	3.74 (0.72)	3.87 (0.74)	**1.828 (1.494 to 2.238)**	**1.834 (1.481 to 2.272)**	**< 0.001**
EQ-5D-5L index score (SD)^j^	0.81 (0.146)	0.75 (0.174)	0.69 (0.190)	0.63 (0.233)	**0.060 (0.028 to 0.128)**	**0.049 (0.022 to 0.109)**	**< 0.001**

Statistically significant (*p* < 0.05) values are indicated in bold.

^a^ Ordinal logistic regression.

^b^ Ordinal logistic regression adjusted for age, gender, and the number of comorbidities.

^c^ Number of long-term medical conditions, including hypertension, any of the 12 conditions listed in the survey, and all other conditions listed in the patient’s medical records. Conditions within a cluster of similar risk factors and treatment methods (such as asthma and COPD) were counted only once.

^d^ Number of regularly consumed medications based on patient self-reported data and medical records.

^e^ The 12 most frequently self-reported conditions. The reference group consists of individuals without the disorder (for example, the reference group for individuals with osteoporosis consists of individuals without osteoporosis).

^f^ Scores based on GAD-7 questionnaire [23].

^g^ Scores based on PHQ-9 questionnaire [24].

^h^ The scores of health measures are regarded as continuous variables and; therefore, odds ratio values represent the odds for an increase or decrease of the treatment burden category level for a one-unit increase in the health measure score.

^i^ Score based on the answers to a single question ‘How would you rate your health?:’ Excellent (1), Very good (2), Good (3), Fair (4), Poor (5).

^j^ Score based on EQ-5D-5L index score [17].

### Finalising the MTBQ translation

After consulting with the healthcare expert group, a decision was made to use the 11-question MTBQ translation without the high-floor response questions 9 and 10 as the final version of the Lithuanian questionnaire.

## Discussion

### Main findings

The study’s main findings suggest that that the 11-question Lithuanian translation of the Multimorbidity Treatment Burden Questionnaire is suitable for assessing the treatment burden of patients with multimorbidity in Lithuania. It has high internal reliability, good construct validity, high factor dimensionality, and meets the standards of ISOQOL [19]. The Lithuanian translation of the MTBQ is consistent with other MTBQ versions published internationally. The study results show the association between higher treatment burden scores and living in a rural area.

The final proposed version of the Lithuanian MTBQ contains 11 question items as questions items 9 and 10 from the original 13-item questionnaire were excluded because of a high proportion of ‘does not apply’ responses. The 11-item questionnaire has good reliability indicated by the Cronbach alpha value of 0.711 and good inter-item correlation ranges (*r* = 0.15-0.50) between questionnaire items. The observed associations between the overall MTBQ scores and the scores of EQ-5D-5L quality-of-life questionnaire and self-rated health support the construct validity of the questionnaire. Subjects with higher MTBQ treatment burden scores were found to have more comorbidities, worse self-assigned life quality and worse self-rated health scores, as predicted by earlier studies that identified connections between the higher number of comorbidities, treatment burden and deteriorating life quality [3, 4]. The dimensionality analysis indicates that the question items are suitable for factor reduction. The high Kaiser-Meyer Olkin value (0.773) and the statistically significant Bartlett test of sphericity result (*p* < 0.001) indicated sampling adequacy and were close to the values obtained in the previous validation studies [12, 14]. The factor analysis of the question items suggested a three-factor solution, similar to two of the previous MTBQ validation studies [14, 15]. The results showed that the Lithuanian version of MTBQ has high internal reliability, good construct validity and factor dimensionality.

The examination of the modified logistic regression analysis (partial proportional odds model) revealed, similarly to previous studies, that subjects had a higher treatment burden if they had more comorbidities [12, 13, 24], higher anxiety and depression levels [9, 10, 12, 13], a lower EQ-5D-5L quality of life score [18], and had certain long-term disorders, such as heart failure and kidney disease (Table 5) [13, 25]. Our study demonstrates that patients with multimorbidity have significantly higher treatment burden scores if they are receiving polypharmacy, i.e. regularly consume more than five medications or reside in rural areas. We suggest that patients with multimorbidity from rural areas experience a higher treatment burden than urban residents because of the poorer regional accessibility of medical healthcare resources [26].

MTBQ treatment burden measurement tool demonstrates applicability in the context of healthcare systems in Central-Eastern Europe. Our study shows that Lithuanian patients with multimorbidity have average treatment burden scores similar to or higher than those of participants in previous MTBQ validation studies: a large proportion of study participants had a medium treatment burden (25%) and high treatment burden (9%) (Table 5) [12, 13]. The study results show significant negative associations between the scores of life quality and general health questionnaires observed in the previous MTBQ validation studies [12, 13]. The Lithuanian MTBQ validation study findings prove that MTBQ applies to different European populations [12, 24]. Further research is now needed to provide insights into the treatment burden effects on the life quality of the patients and their life priorities [27, 28].

## Conclusion

Our study findings demonstrate that the 11-item Lithuanian version of MTBQ has good content and construct validity, reliability, and interpretability and is suitable for assessing treatment burden for patients with multimorbidity in Lithuania. The questionnaire items have appropriate inter-item correlations for most questions and can be reduced into a 3-factor solution. The socio-demographic analysis of our MTBQ translation indicates a positive relationship between living in a rural area and higher treatment burden scores. It also suggests that Lithuanian patients with multimorbidity have average treatment burden scores similar to or higher than participants in previous MTBQ validation studies.
